# Dynamic Thyroglobulin Ratio as a Biomarker to Identify Papillary Thyroid Cancer Patients Who Would Benefit from a Low-Iodine Diet

**DOI:** 10.3390/diagnostics16030456

**Published:** 2026-02-01

**Authors:** Su Woong Yoo, Yong Min Na, Young Jae Ryu, Hee Kyung Kim, Hyun-Jung Choi, Seong-Young Kwon

**Affiliations:** 1 Department of Nuclear Medicine, Chonnam National University Medical School and Hwasun Hospital, Hwasun-gun 58128, Jeonnam, Republic of Korea; 2 Department of Surgery, Chonnam National University Medical School and Hwasun Hospital, Hwasun-gun 58128, Jeonnam, Republic of Korea; 3 Department of Internal Medicine, Chonnam National University Medical School and Hwasun Hospital, Hwasun-gun 58128, Jeonnam, Republic of Korea; 4 Department of Laboratory Medicine, Chonnam National University Medical School and Hospital, Gwangju 61469, Republic of Korea

**Keywords:** papillary thyroid carcinoma, low-iodine diet, radioactive iodine, thyroglobulin

## Abstract

**Objectives**: This study aimed to assess whether low-iodine diet (LID) adherence is associated with therapeutic response in papillary thyroid carcinoma (PTC), specifically in relation to post-therapeutic thyroglobulin (Tg) release as a surrogate marker for the acute radiation-induced response following radioactive iodine (RAI) therapy. **Methods**: This retrospective study included 895 patients with PTC treated with RAI. LID adherence was assessed using the urine iodine-to-creatinine (I/Cr) ratio, with <66.2 μg/g Cr defined as good adherence. The Tg ratio (ratioTg), calculated by dividing post-RAI Tg (measured 7 days after RAI) by pre-RAI Tg, was used to reflect the magnitude of the radiation-induced Tg release. Patients were stratified by ratioTg (≤1 vs. >1), and associations between LID adherence and therapeutic response were analyzed within each group. **Results**: Well-adherent patients exhibited significantly higher ratioTg compared to poorly adherent patients (15.7 ± 2.2 vs. 8.9 ± 1.3, *p* = 0.007). Among patients with ratioTg > 1 (*n* = 630), LID adherence was independently associated with improved therapeutic response (OR, 2.004; 95% CI, 1.270–3.162; *p* = 0.003). No such association was observed in patients with ratioTg ≤ 1 (*n* = 265; *p* = 0.546). **Conclusions**: The clinical benefit of LID appears to depend on the presence of a certain magnitude of radiation-induced Tg release. RatioTg may serve as a useful marker for identifying patients likely to benefit from LID.

## 1. Introduction

Differentiated thyroid carcinoma (DTC), including papillary and follicular subtypes, is one of the most prevalent endocrine cancers, with its incidence steadily increasing worldwide [1]. The prognosis of DTC is generally favorable, reflecting both its indolent biology and the effectiveness of standard treatments, including surgery, radioactive iodine (RAI) therapy, and thyroid-stimulating hormone (TSH) suppression, in appropriately selected patients [2].

RAI therapy plays a pivotal role in the management of DTC by exploiting the thyroid follicular cells’ unique ability to uptake and retain iodine [3,4]. This targeted approach has been shown to reduce the risk of recurrence in selected patients, particularly those with intermediate- or high-risk disease [5,6]. However, the therapeutic efficacy of RAI is influenced not only by the administration protocol, but also by individual patient factors, including iodine status and tumor biology [7].

A low-iodine diet (LID) is typically recommended for 1–2 weeks prior to RAI therapy to reduce the body’s iodine pool and enhance RAI uptake by thyroid cells [3,8]. Despite its routine use, the clinical impact of LID adherence on treatment outcomes remains controversial [9]. Some studies have shown that strict adherence to LID improves the therapeutic response, whereas others have found no significant benefit, highlighting the need to determine the specific biological conditions under which LID exerts a meaningful effect.

Serum thyroglobulin (Tg), a glycoprotein produced by normal and neoplastic thyroid follicular cells, is a well-established biomarker for monitoring disease burden and response to therapy in DTC [10,11,12]. Serum Tg levels measured before RAI therapy primarily reflect the burden of residual thyroid tissue or metastatic disease, whereas it is hypothesized that a transient rise in Tg levels after RAI administration may serve as a surrogate marker reflecting the acute biological response of thyroid tissue to radiation [13].

LID adherence may enhance treatment response under specific conditions, such as when a certain degree of acute radiation-induced response occurs. In this study, we investigated whether LID adherence is associated with therapeutic benefit in such cases, using post-RAI Tg elevation as a surrogate marker of radiation-induced Tg release in patients with papillary thyroid carcinoma (PTC).

## 2. Materials and Methods

### 2.1. Patients

We initially included 1102 patients with PTC who underwent total thyroidectomy followed by the first RAI therapy between 2014 and 2019. Patients were aged ≥ 18 years and had no gross residual disease or distant metastasis before RAI therapy. All patients were instructed to follow a standardized LID for two weeks prior to RAI therapy, which included avoidance of iodine-rich foods such as seaweed, seafood, dairy products, and iodized salt. To elevate TSH, recombinant human TSH (rhTSH) or thyroid hormone withdrawal (THW) was applied before RAI therapy. The exclusion criteria were as follows: the presence of other primary malignancies at the time of diagnosis (*n* = 27); a time interval between surgery and RAI therapy of less than two weeks or more than 12 months (*n* = 2); serum anti-Tg antibody (TgAb) levels greater than 100 U/mL (*n* = 82); loss to follow-up (*n* = 16); and RAI dose of 2.96 GBq (*n* = 80). In total, 895 PTC patients were retrospectively enrolled in this study. This retrospective study was approved by the institutional review board of our hospital in accordance with the Declaration of Helsinki (Approval No. CNUHH-2021-043), and the informed consent was waived.

### 2.2. Measurement of Clinical Parameters at the Time of RAI Therapy or Follow-Up

Serum Tg levels were measured twice: immediately before RAI therapy (pre-RAI Tg) and 7 days after RAI therapy (post-RAI Tg). TgAb and TSH levels were also evaluated prior to RAI therapy. Tg, TgAb, and TSH levels were measured using an immunoradiometric assay (IRMA) with a lower detection limit of <1 ng/mL (RIA Tg-plus, BRAHMS GmbH, Hennigsdorf, Germany), a radioimmunoassay (RIA anti-Tgn, BRAHMS GmbH, Hennigsdorf, Germany), and an IRMA with a lower detection limit of 0.07 µIU/mL (TSH-CTK-3, DiaSorin, Saluggia, Italy), respectively.

Urine iodine and creatinine levels were measured immediately before RAI therapy. Urine iodine concentration was quantified using the AutoLab Iodine assay (IVD-LAB, Hwaseong-si, Gyeonggi, Republic of Korea) on a clinical chemistry analyzer (AU5822, Beckman Coulter Inc., Brea, CA, USA) utilizing the Sandell–Kolthoff reaction. Urine creatinine levels were determined on the same AU5822 analyzer using the CREATININE assay (Beckman Coulter Inc., Brea, CA, USA), based on the compensated kinetic Jaffe method, traceable to the isotope dilution mass spectrometry (IDMS) method. The urine iodine-to-creatinine (I/Cr) ratio (μg/g Cr) was then calculated.

Response assessments using serum Tg and neck ultrasound (US) were performed between 6 and 24 months after the initial RAI therapy [3]. Serum Tg was measured via electrochemiluminescent immunoassay (ECLIA) (Elecsys Tg II, Roche Diagnostics, Penzberg, Germany) using the Cobas e801 module (Roche Diagnostics, Mannheim, Germany). Additionally, TgAb (Elecsys Anti-Tg, Roche Diagnostics, Penzberg, Germany) and TSH (Elecsys TSH, Roche Diagnostics, Penzberg, Germany) levels were measured using the same e801 module on the same day as the Tg measurement.

### 2.3. Study Design

To investigate whether LID adherence is associated with therapeutic benefit, particularly when a certain degree of acute radiation-induced response occurred, we evaluated ratioTg changes according to LID status, its validity as a biomarker, and the resulting conditional efficacy of LID.

We examined whether LID adherence could influence the extent of RAI-induced Tg release. LID adherence was assessed using the urine I/Cr ratio, with a cut-off value of 66.2 μg/g Cr, as established in a previous study conducted in Korea [14]. Patients with a urine I/Cr ratio < 66.2 μg/g Cr were classified as well-adherent, whereas those with a urine I/Cr ratio ≥ 66.2 μg/g Cr were classified as poorly adherent, serving as an operational definition of dietary compliance in this study. The degree of RAI-induced Tg release was indirectly evaluated using the change in serum Tg levels before and after RAI therapy [15,16], expressed as the ratio of post-RAI to pre-RAI Tg levels (ratioTg). We compared ratioTg values between well-adherent and poorly adherent groups to determine whether LID was associated with greater thyroid cell response.

Next, we investigated whether the association between LID adherence and therapeutic response varied depending on the extent of Tg release. Therapeutic response was assessed based on the 2015 American Thyroid Association (ATA) guidelines: (1) excellent response (ER), defined as negative imaging and suppressed Tg < 0.2 ng/mL or TSH-stimulated Tg < 1 ng/mL; (2) indeterminate response (IR), characterized by non-specific findings on imaging studies or suppressed Tg that is detectable but < 1 ng/mL, or stimulated Tg between 1 and 10 ng/mL; (3) biochemical incomplete response (BIR), indicated by negative imaging with suppressed Tg ≥ 1 ng/mL, TSH-stimulated Tg ≥ 10 ng/mL, or increased TgAb values; and (4) structural incomplete response (SIR), identified as structural or functional evidence of disease regardless of Tg level [3]. Patients were categorized into the ER and the non-ER (nER) groups, with the nER group including patients with IR, BIR, and SIR. Patients were stratified into two groups based on a ratioTg threshold of 1.0, under the assumption that a ratioTg > 1 indicates a certain degree of RAI-induced tissue response. Within each group, the association between LID adherence and therapeutic response (ER vs. nER) was then evaluated.

Lastly, we evaluated whether the association between ratioTg and therapeutic response differed depending on LID adherence. This aimed to explore whether ratioTg functions as a biologically meaningful parameter that links LID adherence to therapeutic outcomes, thereby supporting its role as a surrogate marker of RAI-induced thyroid tissue response.

### 2.4. Statistical Analysis

Quantitative data were presented as mean ± standard deviation (SD), or standard error of the mean (SEM), while qualitative data were presented as percentages. Differences in variables were evaluated using *t*-tests for continuous variables and chi-square tests for categorical variables. Multivariate logistic regression analysis was performed to identify factors predicting ER and nER. Variables for multivariate logistic regression were selected based on a combination of univariate analysis results and established clinical relevance. *p*-values less than 0.05 were considered statistically significant. All statistical analyses were conducted using IBM SPSS Statistics for Windows, version 29.0.2.0 (IBM Corp., Armonk, NY, USA).

## 3. Results

### 3.1. Patient Characteristics

Table 1 summarizes the baseline characteristics of the 895 patients included in this study. The mean age was 49.1 ± 12.5 years (range, 18–83 years), and 74.2% were female. All patients had TSH levels greater than 30 µIU/mL. Tumor staging revealed that most patients were classified as T1 (65.2%) or T3 (20.0%), followed by T4 (8.5%) and T2 (6.3%). Lymph node status was categorized as N0a/N0b (20.8%), N1a (55.0%), and N1b (24.2%). The mean diameter of the largest tumor was 1.3 ± 0.9 cm (range, 0.1–7.0 cm). Tumor multiplicity was evenly distributed, with 45.5% of patients having solitary tumors and 54.5% having multiple tumors. Gross extrathyroidal extension was present in 27.4% of cases. The mean number of metastatic lymph nodes per patient was 4.3 ± 5.2. The mean pre-RAI Tg level was 2.6 ± 0.4 ng/mL (range, 0.1–308.0 ng/mL), and the mean value of ratioTg was 13.4 ± 1.5 (range, 0.007–1004.0). Most patients (81.1%) received rhTSH for stimulation, while 18.9% underwent THW.

LID adherence was assessed using the urine I/Cr ratio; 66.1% of patients (*n* = 592) had a ratio < 66.2 μg/g Cr, indicating good adherence, whereas 33.9% (*n* = 303) had a ratio ≥ 66.2 μg/g Cr, indicating poor adherence.

### 3.2. Comparison of ratioTg Values According to Low Iodine Diet Adherence

**Figure 1:**
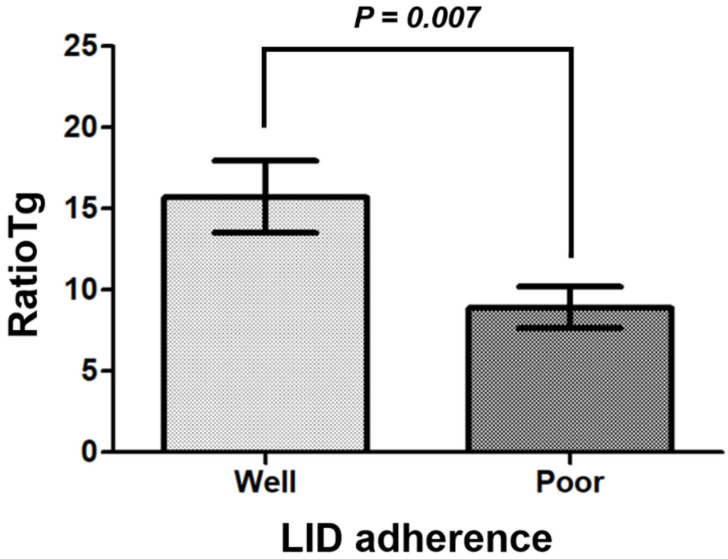
Comparison of ratioTg values according to low iodine diet (LID) adherence. The well-adherent LID group exhibited significantly higher ratioTg levels (15.7 ± 2.2) compared to the poorly adherent LID group (8.9 ± 1.3). The graph represents mean ± standard error of the mean.

The serum pre-RAI Tg level, reflecting residual functioning thyroid tissue after surgery, did not significantly differ between the well-adherent (2.8 ± 0.6 ng/mL) and poorly adherent LID groups (2.2 ± 0.5 ng/mL; *p* = 0.754). To evaluate the impact of LID adherence on RAI-induced thyroid tissue response, we compared ratioTg values between groups. As shown in Figure 1, the well-adherent group had a significantly higher ratioTg (15.7 ± 2.2) than the poorly adherent group (8.9 ± 1.3; *p* = 0.007). There was no significant difference in RAI dose between the groups (*p* = 0.890), allowing us to exclude the effect of RAI dose as a confounding factor in the observed difference in thyroid tissue response.

### 3.3. Factors Associated with RAI Response in All Patients

Among the enrolled patients, 677 (75.6%) were classified into the ER group and 218 (24.4%) into the nER group (Table 1). We analyzed the factors associated with RAI response in the entire patient group (Table 2). In the multivariate logistic regression analysis, female sex (odds ratio [OR], 1.710; 95% confidence interval [CI], 1.150–2.544; *p* = 0.008), smaller tumor size (OR, 1.766; 95% CI, 1.192–2.617; *p* = 0.005), solitary tumor (OR, 1.653; 95% CI, 1.134–2.408; *p* = 0.009), number of metastatic lymph nodes (OR, 1.658; 95% CI, 1.035–2.659; *p* = 0.036), and pre-RAI Tg levels (OR, 1.588; 95% CI, 1.422–1.774; *p* < 0.001) were significantly associated with achieving ER. However, ratioTg (*p* = 0.200) and LID adherence (*p* = 0.177) were not significant predictors in the overall group.

### 3.4. Association Between LID Adherence and Therapeutic Response According to ratioTg

Based on the hypothesis that the therapeutic benefit of LID may emerge when a certain degree of RAI-induced thyroid tissue response has occurred, patients were stratified according to ratioTg (≤1 or >1). Within each group, the association between LID adherence and therapeutic response was assessed. In patients with a ratioTg > 1 (*n* = 630), LID adherence was significantly associated with therapeutic response in the multivariate logistic regression analysis (OR, 2.004; 95% CI, 1.270–3.162; *p* = 0.003) (Table 3). Tumor size (OR, 2.041; 95% CI, 1.278–3.261; *p* = 0.003), solitary tumor (OR, 1.740; 95% CI, 1.125–2.691; *p* = 0.013), and pre-RAI Tg level (OR, 1.705; 95% CI, 1.474–1.973; *p* < 0.001) were also independently associated with therapeutic response in this subgroup. However, in patients with a ratioTg ≤1 (*n* = 265), no significant association was observed between LID adherence and therapeutic response (*p* = 0.546).

### 3.5. Predictive Value of ratioTg in Relation to LID Adherence

**Figure 2:**
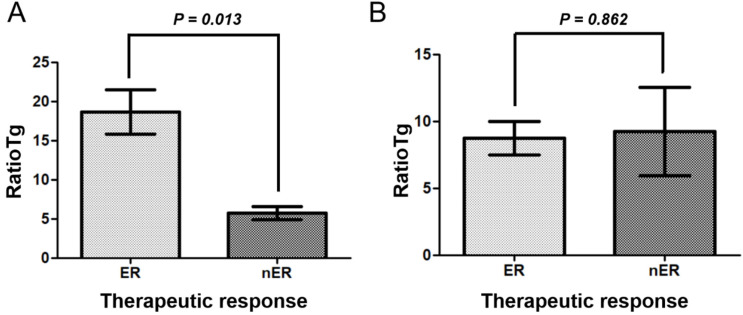
The relationship between therapeutic response and ratioTg in two groups categorized based on low iodine diet (LID) adherence. (**A**) In the well-adherent LID group, the ratioTg levels were significantly higher in the excellent response (ER) group (18.7 ± 2.8) compared to the non-excellent response (nER) group (5.8 ± 0.8) (*p* = 0.013). (**B**) In the poorly adherent LID group, there was no significant difference in ratioTg between the ER group (8.8 ± 1.3) and the nER group (9.3 ± 3.3) (*p* = 0.862). The graph represents mean ± standard error of the mean.

We analyzed the relationship between therapeutic response and ratioTg in two groups stratified by LID adherence (Figure 2). In the well-adherent LID group (Figure 2A), ratioTg levels were significantly higher in the ER group (18.7 ± 2.8) than in the nER group (5.8 ± 0.8) (*p* = 0.013). In multivariate logistic regression analysis, a higher ratioTg was significantly associated with a reduced risk of nER in the well-adherent group (OR, 0.979; 95% CI, 0.961–0.998; *p* = 0.030) (Table 4). Other significant predictors of ER included female sex (OR, 1.745; 95% CI, 1.077–2.826; *p* = 0.024), solitary tumor (OR, 1.675; 95% CI, 1.035–2.711; *p* = 0.036), number of metastatic lymph nodes (OR, 1.928; 95% CI, 1.079–3.446; *p* = 0.027), and pre-RAI Tg (OR, 1.632; 95% CI, 1.420–1.875; *p* < 0.001) (Table 4). In contrast, in the poorly adherent LID group (Figure 2B), there was no significant difference in ratioTg between the ER (8.8 ± 1.3) and nER (9.3 ± 3.3) groups (*p* = 0.862).

Sensitivity analysis excluding extreme ratioTg values yielded consistent results; the clinical benefit of LID in patients with ratioTg > 1.0 and the independent predictive value of ratioTg for treatment response remained statistically significant. These findings confirm that our primary conclusions were not disproportionately influenced by extreme outliers.

## 4. Discussion

In this study, LID adherence, defined by the urine I/Cr ratio, was associated with a significant increase in ratioTg, reflecting enhanced Tg release after RAI therapy. When stratified by a ratioTg threshold of 1, the association between LID adherence and therapeutic response was observed only in patients with ratioTg > 1. Additionally, ratioTg served as a predictive factor for therapeutic response only in the well-adherent LID group.

Although LID is usually recommended for RAI therapy, high-quality studies on its association with therapeutic response are limited, and conflicting results have been reported [9]. One study suggested that in DTC patients, urine iodine excretion exceeding 200 µg/day at the time of RAI therapy was associated with a higher risk of disease progression [17]. In contrast, another prospective study reported that urine iodine excretion did not have predictive value for therapeutic outcomes [18,19]. Our study also found no significant association between LID adherence and therapeutic response in the entire patient cohort (*p* = 0.177 in Table 2).

While LID is widely used as a preparatory step to enhance RAI uptake, its clinical efficacy depends on whether the accumulated RAI can effectively induce thyroid cell destruction. In other words, reducing iodine pool through LID may increase intracellular RAI concentration, but this only translates into therapeutic benefit when thyroid cells are sufficiently susceptible to radiation-induced damage [20]. Our findings further support this concept. Among patients with poor therapeutic response (nER), the ratioTg in the well-adherent LID group (5.8 ± 0.8) tended to be lower than that in the poorly adherent LID group (9.3 ± 3.3) (Figure 2). This suggests that even when iodine uptake is optimized through LID, effective cell destruction may not occur if the biological conditions are unfavorable. Thus, LID alone cannot guarantee improved outcomes, and its impact should be evaluated in the context of whether thyroid cells can actually be destroyed by RAI. These findings highlight the need for more precise criteria or biomarkers to identify patients who are likely to benefit from LID.

Following RAI therapy, Tg stored within thyroid follicular cells is passively released into the bloodstream due to radiation-induced membrane disruption and apoptosis. This results in a transient rise in serum Tg levels [13], which can indirectly reflect the extent of tissue destruction. Several prior studies have explored the clinical relevance of this phenomenon by examining the ratio between pre- and post-RAI Tg levels (ratioTg), a metric that normalizes for baseline variations across different TSH stimulation methods, demonstrating that higher ratioTg values are associated with better short-term outcomes [15,16]. These findings support the use of ratioTg as a surrogate marker for RAI-induced thyroid tissue response and, by extension, a context in which LID adherence could meaningfully influence therapeutic results. Building on this rationale, we employed ratioTg in our study to stratify patients and evaluate the association between LID adherence and therapeutic response in a biologically informed manner.

In our study, we stratified patients based on a ratioTg threshold of 1.0 to examine the potential therapeutic relevance of LID adherence under varying degrees of RAI-induced thyroid tissue response. This decision was based on the hypothesis that LID contributes to treatment efficacy only when RAI induces a certain degree of RAI-induced thyroid tissue response that leads to Tg release due to loss of cell membrane integrity. A ratioTg greater than 1 reflects a net increase in serum Tg following RAI therapy, which can reasonably be interpreted as a surrogate for acute tissue response. While this biologically informed threshold provides a practical framework for analysis, it should be noted that the cutoff value of 1.0 has not been universally validated. The selection was intended to distinguish between patients with minimal and those with appreciable Tg release. Further studies are warranted to refine this threshold and to determine whether alternative cutoffs or continuous evaluation of Tg changes could more accurately reflect the biologically relevant extent of thyroid tissue response.

These results have several implications. They suggest that the effect of LID should not be assessed simply through a binary comparison with treatment response, but rather within the biological context of thyroid tissue response following RAI therapy. RatioTg may serve as a surrogate marker to identify the conditions under which LID is likely to confer therapeutic benefit. The therapeutic benefit of LID was significant only in patients with elevated ratioTg levels, indicating that even with adequate LID adherence, additional therapeutic strategies may be necessary for patients exhibiting limited Tg release after RAI therapy. Nonetheless, caution is warranted in making direct causal interpretations, as ratioTg represents an intermediate post-intervention marker that may be subject to mediation or collider bias.

This study has several limitations. First, its retrospective design may introduce selection bias, though the inclusion of a relatively large number of patients may help reduce this concern. Second, the ratioTg is influenced by a complex interplay of radiation-induced follicle damage, transient inflammation, and Tg leakage. A single-point measurement on day 7 may not fully capture the entire kinetic profile of this response, which may vary between normal remnants and metastatic tissues. Further studies are needed to better understand how these temporal dynamics can be leveraged to more accurately reflect the magnitude of the acute biological response to RAI. In addition, the ratioTg cutoff of 1.0 was selected to distinguish between minimal and measurable Tg release following RAI therapy, rather than to optimize predictive performance. Further studies are warranted to refine this threshold and validate its clinical utility. Lastly, the binary classification (ER vs. nER) may obscure subgroup heterogeneity, and this study focused on short-term treatment outcomes. Future research with long-term follow-up is needed to assess the prognostic relevance of LID adherence under specific conditions, such as those indicated by ratioTg.

## 5. Conclusions

LID adherence was associated with increased Tg release following RAI, as reflected by higher ratioTg values. However, the therapeutic benefit of LID was observed only in patients with ratioTg > 1, suggesting that LID enhances therapeutic efficacy primarily in cases where a certain magnitude of radiation-induced Tg release is achieved. RatioTg may serve as a useful indicator for identifying patients likely to benefit from LID adherence.

## Figures and Tables

**Table 1:** Patient characteristics (*n* = 895).

Variables	Values
Age (years)	
Mean ± SD (range)	49.1 ± 12.5 (18–83)
Sex	
Male	231 (25.8%)
Female	664 (74.2%)
T stage	
T1	584 (65.2%)
T2	56 (6.3%)
T3	179 (20.0%)
T4	76 (8.5%)
N stage	
N0a or N0b	186 (20.8%)
N1a	492 (55.0%)
N1b	217 (24.2%)
Diameter of the largest tumor (cm)	
Mean ± SD (range)	1.3 ± 0.9 (0.1–7.0)
Multiplicity	
Solitary	407 (45.5%)
Multiple	488 (54.5%)
Gross extrathyroidal extension	
No	650 (72.6%)
Yes	245 (27.4%)
Number of metastatic lymph nodes	
Mean ± SD (range)	4.3 ± 5.2 (0–36)
RAI dose	
Low does (≤1.85 GBq)	452 (50.5%)
High does (≥3.70 GBq)	443 (49.5%)
Pre-RAI Tg (ng/mL)	
Mean ± SEM (range)	2.6 ± 0.4 (0.1–308.0)
RatioTg	
Mean ± SEM (range)	13.4 ± 1.5 (0.007–1004.0)
Preparation method	
THW	169 (18.9%)
rhTSH	726 (81.1%)
Urine I/Cr ratio (μg/g Cr)	
<66.2	592 (66.1%)
≥66.2	303 (33.9%)
Response assessment	
ER	677 (75.6%)
nER	218 (24.4%)

SD, standard deviation; RAI, radioactive iodine; Tg, thyroglobulin; SEM, standard error of the mean; THW, thyroid hormone withdrawal; rhTSH, recombinant human thyrotropin; ER, excellent response; nER, non-excellent response.

**Table 2:** Factors associated with therapeutic response in the overall study population.

Variables	ER (*n* = 677)	nER (*n* = 218)	Univariate	Multivariate	
			*p* Value	*p* Value	Odds Ratio (95% CI)
Age (years)			0.971		
<55 years	445 (75.7%)	143 (24.3%)			
≥55 years	232 (75.6%)	75 (24.4%)			
Sex			< 0.001	0.008	1.710 (1.150–2.544)
Male	150 (64.9%)	81 (35.1%)			
Female	527 (79.4%)	137 (20.6%)			
T stage			0.004	0.992	1.009 (0.175–5.832)
T1 + T2	501 (78.3%)	139 (21.7%)			
T3 + T4	176 (69.0%)	79 (31.0%)			
N stage			<0.001	0.238	1.235 (0.870–1.755)
N0a, N0b	163 (87.6%)	23 (12.4%)			
N1a	385 (78.3%)	107 (21.7%)			
N1b	129 (59.4%)	88 (40.6%)			
Tumor size (cm)			< 0.001	0.005	1.766 (1.192–2.617)
≤1.0	334 (81.9%)	74 (18.1%)			
>1.0	343 (70.4%)	144 (29.6%)			
Multiplicity			< 0.001	0.009	1.653 (1.134–2.408)
Solitary	334 (82.1%)	73 (17.9%)			
Multiple	343 (70.3%)	145 (29.7%)			
Gross extrathyroidal extension			0.004	0.671	1.469 (0.250–8.648)
No	508 (78.1%)	142 (21.9%)			
Yes	169 (69.0%)	76 (31.0%)			
Number of metastatic lymph nodes			< 0.001	0.036	1.658 (1.035–2.659)
≤5	536 (82.3%)	115 (17.7%)			
>5	141 (57.8%)	103 (42.2%)			
RAI dose (GBq)			0.001	0.842	1.040 (0.706–1.532)
Low dose (≤1.85 GBq)	364 (80.5%)	88 (19.5%)			
High dose (≥3.7 GBq)	313 (70.7%)	130 (29.3%)			
Pre-RAI Tg (ng/mL)			< 0.001	< 0.001	1.588 (1.422–1.774)
Mean ± SEM	0.8 ± 0.1	8.3 ± 1.7			
RatioTg			< 0.001	0.200	0.994 (0.985–1.003)
Mean ± SEM	15.5 ± 1.9	7.1 ± 1.3			
Urine I/Cr ratio (μg/g Cr)			0.177		
<66.2	456 (77.0%)	136 (23.0%)			
≥66.2	221 (72.9%)	82 (27.1%)			
Preparation			0.407		
THW	132 (78.1%)	37 (21.9%)			
rhTSH	545 (75.1%)	181 (24.9%)			

ER, excellent response; nER, non-excellent response; RAI, radioactive iodine; Tg, thyroglobulin; SEM, standard error of the mean; THW, thyroid hormone withdrawal; rhTSH, recombinant human thyrotropin.

**Table 3:** Factors associated with therapeutic response in patients with a ratioTg > 1.

Variables	ER (*n* = 474)	nER (*n* = 156)	Univariate	Multivariate	
			*p* Value	*p* Value	Odds Ratio (95% CI)
Age (years)			0.590		
<55 years	321 (75.9%)	102 (24.1%)			
≥55 years	153 (73.9%)	54 (26.1%)			
Sex			0.003	0.068	1.558 (0.967–2.509)
Male	117 (66.9%)	58 (33.1%)			
Female	357 (78.5%)	98 (21.5%)			
T stage			0.014	0.990	0.989 (0.172–5.675)
T1 + T2	347 (78.0%)	98 (22.0%)			
T3 + T4	127 (68.6%)	58 (31.4%)			
N stage			< 0.001	0.459	1.168 (0.774–1.762)
N0a, N0b	109 (85.8%)	18 (14.2%)			
N1a	276 (77.5%)	80 (22.5%)			
N1b	89 (60.5%)	58 (39.5%)			
Tumor size (cm)			< 0.001	0.003	2.041 (1.278–3.261)
≤1.0	228 (83.5%)	45 (16.5%)			
>1.0	246 (68.9%)	111 (31.1%)			
Multiplicity			< 0.001	0.013	1.740 (1.125–2.691)
Solitary	245 (81.7%)	55 (18.3%)			
Multiple	229 (69.4%)	101 (30.6%)			
Gross extrathyroidal extension			0.013	0.798	1.262 (0.213–7.486)
No	353 (77.9%)	100 (22.1%)			
Yes	121 (68.4%)	56 (31.6%)			
Number of metastatic lymph nodes			< 0.001	0.057	1.719 (0.984–3.003)
≤5	377 (81.3%)	87 (18.8%)			
>5	97 (58.4%)	69 (41.6%)			
RAI dose (GBq)			0.004	0.827	0.949 (0.596–1.512)
Low dose (≤1.85 GBq)	242 (80.4%)	59 (19.6%)			
High dose (≥3.7 GBq)	232 (70.5%)	97 (29.5%)			
Pre-RAI Tg (ng/mL)			< 0.001	<0.001	1.705 (1.474–1.973)
Mean ± SEM	0.8 ± 1.3	5.2 ± 10.4			
Urine I/Cr ratio (μg/g Cr)			0.045	0.003	2.004 (1.270–3.162)
<66.2	327 (77.7%)	94 (22.3%)			
≥66.2	147 (70.3%)	62 (29.7%)			
Preparation			0.507		
THW	103 (77.4%)	30 (22.6%)			
rhTSH	371 (74.6%)	126 (25.4%)			

ER, excellent response; nER, non-excellent response; RAI, radioactive iodine; Tg, thyroglobulin; SEM, standard error of the mean; THW, thyroid hormone withdrawal; rhTSH, recombinant human thyrotropin.

**Table 4:** Factors associated with therapeutic response in well-adherent low-iodine diet group.

Variables	ER (*n* = 456)	nER (*n* = 136)	Univariate	Multivariate	
			*p* Value	*p* Value	Odds Ratio (95% CI)
Age (years)			0.740		
<55 years	334 (76.6%)	102 (23.4%)			
≥55 years	122 (78.2%)	34 (21.8%)			
Sex			< 0.001	0.024	1.745 (1.077–2.826)
Male	114 (66.3%)	58 (33.7%)			
Female	342 (81.4%)	78 (18.6%)			
T stage			0.124		
T1 + T2	340 (78.7%)	92 (21.3%)			
T3 + T4	116 (72.5%)	44 (27.5%)			
N stage			< 0.001	0.669	1.102 (0.705–1.723)
N0a, N0b	97 (89.0%)	12 (11.0%)			
N1a	269 (79.6%)	69 (20.4%)			
N1b	90 (62.1%)	55 (37.9%)			
Tumor size (cm)			0.062		
≤1.0	213 (80.7%)	51 (19.3%)			
>1.0	243 (74.1%)	85 (25.9%)			
Multiplicity			0.004	0.036	1.675 (1.035–2.711)
Solitary	218 (82.6%)	46 (17.4%)			
Multiple	238 (72.6%)	90 (27.4%)			
Gross extrathyroidal extension			0.118		
No	346 (78.6%)	94 (21.4%)			
Yes	110 (72.4%)	42 (27.6%)			
Number of metastatic lymph nodes			< 0.001	0.027	1.928 (1.079–3.446)
≤5	356 (83.2%)	72 (16.8%)			
>5	100 (61.0%)	64 (39.0%)			
RAI dose (GBq)			0.171		
Low dose (≤1.85 GBq)	237 (79.5%)	61 (20.5%)			
High dose (≥3.7 GBq)	219 (74.5%)	75 (25.5%)			
Pre-RAI Tg (ng/mL)			< 0.001	< 0.001	1.632 (1.420–1.875)
Mean ± SEM	0.8 ± 0.1	9.5 ± 2.5			
RatioTg			0.013	0.030	0.979 (0.961–0.998)
Mean ± SEM	18.7 ± 2.8	5.8 ± 0.8			
Preparation			0.488		
THW	108 (79.4%)	28 (20.6%)			
rhTSH	348 (76.3%)	108 (23.7%)			

ER, excellent response; nER, non-excellent response; RAI, radioactive iodine; Tg, thyroglobulin; SEM, standard error of the mean; THW, thyroid hormone withdrawal; rhTSH, recombinant human thyrotropin.

## Data Availability

The data presented in this study are available on request from the corresponding author due to privacy and ethical restrictions.
